# Factors affecting poor sleep quality in last trimester pregnant women: a cross-sectional research from Turkey

**DOI:** 10.1590/1806-9282.20240180

**Published:** 2024-09-16

**Authors:** Sibel Peksoy Kaya, Filiz Aslantekin Özçoban, Berna Dilbaz

**Affiliations:** 1 Ankara Yıldırım Beyazıt University, Faculty of Health Sciences, Department of Nursing – Ankara, Turkey.; 2 Balıkesir University, Faculty of Health Sciences, Department of Midwifery – Balıkesir, Turkey.; 3 University of Heath Sciences, Ankara Etlik Zübeyde Hanım Women's Health Training and Research Hospital – Ankara, Turkey.

**Keywords:** Fatigue, Blood cell number, Restless leg syndrome, Sleep quality

## Abstract

**OBJECTIVE:**

The aim of the study was to determine the factors affecting poor sleep quality in the last trimester pregnant women.

**METHODS:**

A cross-sectional study was conducted at a tertiary care maternity hospital in Ankara, Turkey. The research was conducted between May and September 2019 with 570 pregnant women in the last trimester. The data were collected through the Personal Information Form, Pittsburgh Sleep Quality Index, International Physical Activity Questionnaire Short Form, Restless Legs Syndrome Form, Brief Fatigue Inventory, and Perceived Stress Scale.

**RESULTS:**

The mean Pittsburgh Sleep Quality Index score of the pregnant women was 5.98±3.31, and 48.9% of them were found to have over five Pittsburgh Sleep Quality Index scores. Hemoglobin levels, income perceptions, smoking habits, attending pregnant schools, experiencing leg pains or cramping, experiencing back, waist, or neck pains, Restless Legs Syndrome, fatigue levels, and perceived stress levels of the pregnant women were found to be important determinants of sleep quality (p<0.05).

**CONCLUSION:**

According to the findings, increasing hemogram levels, attending antenatal education programs, and improving the ability of pregnant women to manage stress are opportunities to improve sleep quality during pregnancy. Careful evaluation of pregnant women in terms of insomnia and affecting factors can be suggested during antenatal follow-up.

## INTRODUCTION

Various anatomical, physiological, and biochemical changes occur during pregnancy^1^. While some pregnant women can tolerate these changes, their daily life activities may be negatively affected in some of them^2^. Pregnant women can often experience problems such as fatigue, frequent urination, nausea, back pain, or restless leg syndrome (RLS). These problems negatively affect sleep quality^2,3^. Sleeping disorders during pregnancy vary between 46% and 89%. Sleep disorders increase significantly, especially in the last trimester^4-7^.

Sleep disorders occurring during pregnancy can be associated with RLS, stress, obesity, iron deficiency, anemia, nutrition, physical activity, fatigue, smoking, alcohol, and socio-demographic characteristics such as age, education, employment status, and income level^7^. These factors that cause sleep problems during pregnancy are often interrelated^2,7,8^. Pınar et al.^9^ state that 12% of women perceive stress during pregnancy. RLS is among the problems associated with sleep, stress, and anemia during pregnancy. RLS is experienced by 10–34% of women, especially in the third trimester of pregnancy^6,8,10^. Some factors that increase RLS development during pregnancy include caffeine consumption, anemia, and iron deficiency^8^. Iron deficiency, which is the most important cause of anemia^11^, is among the strongest risk factors for the development of RLS^6^.

Sleeping disorders during pregnancy can lead to negative health consequences such as preterm birth, gestational diabetes, and a low infant Apgar score^2,6,7,10^. As stated in the literature, problems affecting daily life have a great effect on the pregnancy period. It is important to reduce sleeping disorders and improve sleep quality for physical and mental health during pregnancy^7^. Therefore, to provide quality care in the perinatal period, strategies should be developed to prevent sleep disorders and improve the health of pregnant women^12^. In the context of developed guidelines and strategies, factors affecting these problems during pregnancy should be considered. Although there is some research on sleep quality in the literature^3-5,8,9,12^, there is limited research on RLS, stress, fatigue, physical activity, body mass index, and anemia. In this study, the poor sleep quality of pregnant women in the last trimester and the factors affecting this situation were investigated. Research findings will guide the planning of interventions to identify and improve factors affecting sleep quality.

## METHODS

### Ethical approval

This study was approved by the ethics committee of a public university (No. 229-20.06.2018) and the hospital (No. 799-28.09.2018) where the study was conducted. Pregnant women were given comprehensive information about the purpose of the research, which was conducted in accordance with the Declaration of Helsinki. Written consent was obtained from the pregnant women.

### Study design

This cross-sectional study was carried out between May and September 2019 in pregnant polyclinics of a tertiary care maternity hospital in Ankara, Turkey. The sample size of the research was determined to be 377 pregnant women according to the formula "estimation of sampling number when the target population is known." Power estimation was made after the study by G*Power 3.1.7. The power calculation made for the difference of 0.50 of Pittsburgh Sleep Quality Index (PSQI) used mean (5.98±3.3) based on study sampling was found to be over 94% [alpha=0.05, effect size (d)=0.15]. A total of 570 pregnant and voluntary women aged 18 years and over, having basic reading and comprehension literacy, having no risk in pregnancy, and being in a period of over 36 weeks in the last trimester were included in this research.

### Data collection tools

The research data were collected through six tools. The personal Information Form was developed by a number of researchers^4,8,9,12^ in line with the literature. It consists of questions regarding the factors that will affect the socio-demographic and obstetric characteristics and sleep of pregnant women.

The PSQI is composed of 24 items (Min:0, Max:21). Each item is scored between 0 and 3 points. A PSQI total score of ≤5 indicates that sleep quality is good, and a score >5 indicates that sleep quality is poor^13^. International Physical Activity Questionnaire Short Form (IPAQ-SF) is a criterion for the evaluation of physical activity to perform each activity over 10 min at a time. The amount of oxygen consumed when physical activity is done is expressed as Metabolic Equivalent (MET). According to MET scores, IPAQ-SF is categorized into "inactive," "minimum active," and "very active"^14^. RLS form consists of four questions in a structure to contain the diagnostic criteria of restless legs syndrome. In the event that all the questions in the form are answered as "yes," then RLS is diagnosed^15^. Brief Fatigue Inventory (BFI) evaluates the general fatigue level in the last 24 h and the effect of this fatigue on daily activities. The BFI score of "0" indicates that there is no fatigue, "1–3" indicates that the fatigue is low, "4–6" indicates that the fatigue is moderate, "7–9" indicates that the fatigue is high, and "10" indicates that it is at the highest level^16^. Perceived Stress Scale (PSS) consists of 10 items (Min:10, Max:50). A score of 30 or above indicates that the stress level is high^17^.

### Data analysis

IBM SPSS Statistics 21.0 (IBM Corp., Armonk, NY) was used for statistical analysis. Number, percentage, and chi-square tests were used to evaluate categorical data. A "student's t-test" was used to evaluate the numerical and scale scores. The eta square (*ƞ* ^2^) was calculated for the effect size of each group. In interpreting the effect sizes, 0.01≤*ƞ* ^2^<0.06 "small," 0.06≤*ƞ* ^2^<0.14 "medium," and *ƞ* ^2^≥0.14 "large effect size" were taken into consideration as limit values. The significance score was taken as p<0.05.

## RESULTS

The mean age of the participants was 27.36±5.3 years. The majority of the women stated that they were housewives (83.5%), did not smoke during pregnancy (88.4%), and had a planned pregnancy (73.7%). The gestational week of the participants was 37.1±1.56, and the number of pregnancies was 2.10±1.36. The hemoglobin value of the participants was 11.48±1.29 g/dL. A total of 69.3% of participants stated that they used folic acid supplements, and 76.3% received iron supplements during pregnancy. Only 17% of participants attended pregnant schools. Other features of the participants are included in Table 1.

**Table 1:** Distribution of some socio-demographic and obstetric characteristics of the participants.

Characteristics		±SD
Age (years)		27.36±5.31
Gestational week		37.1±1.56
Number of pregnancies		2.10±1.36
Number of deliveries		0.92±1.08
Number of living children		0.81±0.94
BMI pre-pregnancy (kg/m^2^)		24.42±4.50
BMI in the third trimester (kg/m^2^)		28.97±4.32
Gestational weight gain (kg)		11.94±5.25
Hemoglobin value (g/dL)		11.48±1.29
Planning status of pregnancy		n (%)
	Planned pregnancy	420 (73.7)
	Unplanned pregnancy	150 (26.3)
Educational status		
	Primary and less*	74 (13.0)
	Secondary school	153 (26.8)
	High school	207 (36.3)
	University	136 (23.9)
Employment		
	Housewife	476 (83.5)
	Employed	94 (16.5)
Income status perception		
	High-income status	51 (8.9)
	Middle-income status	353 (61.9)
	Low-income status	166 (29.1)
Place of residence		
	City	498 (87.4)
	Town/village	72 (12.6)
Smoking status during pregnancy		
	Smoking	66 (11.6)
	Not smoking	504 (88.4)
Frequency of receiving antenatal care		
	Between 1 and 9	59 (10.4)
	10 and more	511 (89.6)
Folic acid supplement consumption		
	Never used	98 (17.2)
	Before conception and during pregnancy	77 (13.5)
	During pregnancy	395 (69.3)
Iron supplement consumption		
	Never used	110 (19.3)
	Before conception and during pregnancy	25 (4.4)
	During pregnancy	435 (76.3)
Participation in pregnant education		
	Participated	97 (17.0)
	Not participated	473 (83.0)

* Ten of the participants were literate. SD: standard deviation; BMI: body mass index.

The mean PSQI score of the participants was 5.98±3.31, and 48.9% of them were found to have PSQI scores above 5. Notably, 50.2% of the participants were inactive, and 25.1% of participants had RLS. The mean BFI score of the participants was 4.43±2.31. Notably, 74.4% of them were found stressed. Other scale scores of participants are included in Table 2.

**Table 2:** Distribution of scale scores in pregnant women.

Score averages of scales		±SD
Pittsburgh Sleep Quality Index		5.98±3.31
International Physical Activity Questionnaires-SF		790.23±746.12
Brief Fatigue Inventory		
	General fatigue level	6.04±2.33
	Fatigue level-daily activity	4.43±2.31
Perceived Stress Scale		25.91±5.13
Categories of scales		
Pittsburgh Sleep Quality Index		n (%)
	≤5 good sleep quality	291 (51.1)
	>5 poor sleep quality	279 (48.9)
International Physical Activity Questionnaires-SF		
	Inactive	286 (50.2)
	Minimum active	274 (48.1)
	Very active	10 (1.8)
Brief Fatigue Inventory		
	No fatigue	17 (3.0)
	Very little fatigue	182 (31.9)
	Moderate fatigue	259 (45.4)
	High fatigue	101 (17.7)
	Maximum fatigue	11 (1.9)
Perceived Stress Scale		
	<29 pregnant stressed	430 (75.4)
	≥30 pregnant not stressed	140 (24.6)
Restless Legs Syndrome Form		
	Have restless legs syndrome	143 (25.1)
	Don't have restless legs syndrome	427 (74.9)

SD: standard deviation; SF: short form.

The hemoglobin value was below 11 g/dL in 26.8% of participants with a PSQI score of 5 or below; in those with a PSQI score above 5, it was 36.6% (p<0.05). There was a difference between their middle-income and low-income levels and PSQI categories (p<0.05). While 14.3% of participants with a PSQI score above 5 smoked, pregnant women with a PSQI score of 5 or below had a smoking status of 8.9% (p<0.05). Participants with a PSQI score of five and below were more likely to attend pregnant schools (21.3%) than participants with a PSQI score above five (12.5%) (p<0.05). Percentages of participants with a PSQI score above 5 experiencing leg pain or cramps (83.9%) and back, waist, or neck pain (88.2%) were higher than those with a PSQI score of 5 or below (p<0.05). RLS prevalence was 29.4% in participants with a PSQI score above 5 (p<0.05). Fatigue level in terms of daily activity of the BFI was 5.16±2.23 in participants with a PSQI score above 5, which is higher than participants with a PSQI score of 5 and below (3.73±2.16) (*ƞ* ^2^ *=*0.096, p<0.05). Fatigue level was at a higher or maximum level in 57.7% of the participants with a PSQI score above 5 (p<0.05). The mean PSS score was 26.89±5.08 in participants with a PSQI score above 5, while this mean was 24.97±5.00 in those with a PSQI score of 5 and below (*ƞ* ^2^ *=*0.035, p<0.05). A total of 28.7% of the participants whose PSQI score was above 5 had a PSS score of 30 or above (Table 3).

**Table 3:** Comparison of sleep quality to variables associated with sleep in participants.

Characteristics		PSQI≤5 (n=291)	PSQI>5 (n=279)	Tests	p-value
		±SD	±SD		
Age (years)		27.45±5.46	27.25±5.15	^c^t=0.270	p=0.655
BMI in the third-trimester		28.91±4.24	29.02±4.40	^c^t=−0.303	p=0.762
Number of pregnancies		2.10±1.35	2.16±1.30	^c^t=−0.555	p=0.579
Fatigue level-daily activity		3.73±2.16	5.16±2.23	^c^t=−7.771	p=0.000**
Perceived Stress Scale		24.97±5.00	26.89±5.08	^c^t=−4.551	p=0.000**
Hemoglobin		n (%)	n (%)		
	<11 g/dL hemoglobin	78 (26.8)	102 (36.6)	^d^ *X* ^2^=6.273	p=0.012*
	≥11 g/dL hemoglobin	213 (73.2)	177 (63.4)		
Planning the pregnancy					
	Planned pregnancy	128 (44.0)	111 (39.8)	^d^ *X* ^2^=1.033	p=0.310
	Unplanned pregnancy	163 (56.0)	168 (60.2)		
Income status perception					
	High-income status	27 (9.3)^a^	24 (8.6)^a^	^d^ *X* ^2^=8.529	p=0.014*
	Middle-income status	195 (67.0)^a^	158 (56.6)^b^		
	Low-income status	69 (23.7)^a^	97 (34.8)^b^		
Smoking status during pregnancy					
	Smoking	26 (8.9)	40 (14.3)	^d^ *X* ^2^=4.060	p=0.044*
	Not smoking	265 (91.1)	239 (85.7)		
Participation in pregnant education					
	Participated	62 (21.3)	35 (12.5)	^d^ *X* ^2^=7.742	p=0.005^*^
	Not participated	229 (78.7)	244 (87.5)		
Leg pains or cramps during pregnancy					
	Existent	222 (76.3)	234 (83.9)	^d^ *X* ^2^=5.118	p=0.024^*^
	Non-existent	69 (23.7)	45 (16.1)		
Back, waist, or neck pain during pregnancy					
	Existent	232 (79.7)	246 (88.2)	^d^ *X* ^2^=7.509	p=0.006*
	Non-existent	59 (20.3)	33 (11.8)		
International Physical Activity Questionnaires-SF					
	Inactive	150 (51.5)	136 (48.7)	^d^ *X* ^2^=0.447	p=0.504
	Minimal or very active	141 (48.5)	143 (51.3)		
Brief Fatigue Inventory					
	No fatigue or very little	132 (45.3)^a^	67 (24.0)^b^	^d^ *X* ^2^=38.594	p=0.000**
	Moderate fatigue	125 (43.0)^a^	134 (48.0)^a^		
	High or maximum fatigue	34 (11.7)^a^	78 (28.0)^b^		
Perceived Stress Scale					
	<29 pregnant stressed	231 (79.4)	199 (71.3)	^d^ *X* ^2^=4.988	p=0.026*
	≥30 pregnant not stressed	60 (20.6)	80 (28.7)		
Restless Legs Syndrome Form					
	Have RLS	61 (21.0)	82 (29.4)	^d^ *X* ^2^=5.384	p=0.020*
	Don't have RLS	230 (79.0)	197 (70.6)		

PSQI: Pittsburgh Sleep Quality Index; SD: standard deviation; BMI: body mass index; SF: short form; RLS: restless legs syndrome. ^a,b^The difference in fatigue levels. ^c^Student's t-test. ^d^Pearson chi-square. Significant p-values are bold.

* p<0.05

** p<0.001.

## DISCUSSION

Many women experience significant sleep problems during pregnancy. Poor sleep quality during pregnancy is associated with negative pregnancy outcomes such as preterm delivery, hypertension, cesarean, low birth weight, and Apgar score^7,10^. In this context, problems related to sleep quality during pregnancy are important parameters affecting maternal and fetal health. In the study, the PSQI mean of participants was 5.98±3.31, and the prevalence of poor sleep quality was 48.9%. Kostanoğlu et al.^18^ stated that the average PSQI mean of pregnant women was 6.18±2.97 and their poor sleep quality was 51.9%; Öztürk-Murat et al.^5^ emphasize that the PSQI mean is 7.27±3.18 and the poor sleep quality is 65.7%. These findings suggest that approximately half or more of pregnant women experience significant sleep problems during pregnancy. It is emphasized in the literature that sleep problems increase, especially in the last trimester^4-7^. In the study, it was determined that sleep problems in the last trimester of pregnancy were affected by factors such as low hemoglobin level, perception of medium or low income, smoking, not attending pregnancy schools, experiencing leg pain or cramps, experiencing back, waist, or neck pain, fatigue, stress, and RLS.

In the study, it was found that hemoglobin level, income perception, smoking habit, attending pregnancy school, experiencing back, waist, or neck pain, experiencing leg pain or cramps, RLS, fatigue, and stress are important determinants of sleep quality in pregnant women. It is reported in the literature that low-income pregnant women are at risk of poor sleep quality compared to high-income pregnant women^7,19^. It is thought that, contrary to high-income pregnant women whose living conditions are better, anxiety related to economic difficulties may cause stress and sleep problems in pregnant women. In the study, it was also found that smoking is an effective factor in poor sleep quality during pregnancy. Conlon et al.^20^ emphasize that sleep quality worsens, especially in the third trimester, and that smoking is also an important factor affecting sleep quality. It is stated in the literature that smoking is associated with stress^8^.

Participation in pregnancy schools was associated with higher sleep quality in pregnant women in the study. Akkus et al.^21^ emphasize that the level of knowledge about quality sleep increases in pregnant women who participate in birth preparation training. Because antenatal education programs increase awareness of possible problems related to the pregnancy process, pregnant women gain the ability to cope with difficulties.

In the study, pregnant women with poor sleep quality experienced "leg pain/cramps/back/waist/neck pain" more often. Gündüz and Yıldız^7^ state that complaints about pain in pregnancy are an important factor affecting sleep quality. Problems such as back pain and leg cramps during pregnancy can lead to poor sleep quality.

A hemogram below 11 g/dL was found to be significant with poor sleep quality in the study. It is reported in the literature that iron deficiency and anemia are associated with RLS^2,4,6,8^. Similar to low blood count, one in every four pregnant women experiences RLS, and this finding is significant with poor sleep quality. Almeneessie et al.^22^ reported a relationship between RLS and parity, anemia, diabetes, vitamin D deficiency, and smoking. RLS is also associated with several risk factors, such as gestational hypertension, preeclampsia, and depression that can affect sleep^2,6,7^. Likewise, low hemoglobin value and smoking were found to be associated with poor sleep quality in the study. These results reinforce that the cumulative increase in risks during pregnancy may increase sleep problems.

In the study, fatigue and stress were among the determining factors in terms of sleep quality in pregnant women. Sleep quality during pregnancy is associated with fatigue and mood disorders^6,7,9^. Pınar et al.^9^ reported that there is a relationship between sleep quality and perceived stress and that sleep quality worsens with increasing stress. As sleep problems increase, general fatigue and daily activities may also be negatively affected. Being parents and changing family roles can be a source of stress, especially for pregnant women. It is thought that pregnancy and delivery, which are perceived as stressful life events, may also cause sleep problems.

## CONCLUSION

The research indicates that poor sleep quality is common among pregnant women in the last trimester. Low hemoglobin level, low- or middle-income status, smoking, not getting antenatal education, experiencing back, waist, neck, or leg pains, RLS, fatigue, and stress have all been found to be associated with poor sleep quality. Increasing the level of hemoglobin, active participation in childbirth classes, and improving the ability of pregnant women to manage stress are seen as opportunities to improve sleep quality. In addition to smoking being a factor associated with stress and sleep quality, raising awareness among pregnant women about quitting smoking is also important for a healthy pregnancy. Healthcare professionals should provide preventive and supportive care for sleep problems, evaluate such problems, and refer them to relevant health professionals or institutions when necessary.
